# Editorial: Harnessing the potential of functional foods containing bioactive compounds: implications for health and sustainability

**DOI:** 10.3389/fnut.2026.1797073

**Published:** 2026-02-19

**Authors:** Ali Rashidinejad, Muhammad Ijaz Ahmad

**Affiliations:** 1 Riddet Institute, Massey University, Palmerston North, New Zealand; 2 College of Biosystems Engineering and Food Science, Zhejiang Key Laboratory for Agro-Food Processing, Zhejiang University, Hangzhou, China

**Keywords:** antioxidant activity, bioactive compounds, bioactive delivery systems, functional foods, mechanistic pathways, metabolic health, polyphenols, sustainability

Functional foods containing bioactive compounds are increasingly recognized as critical interfaces between nutrition science, preventive health, and sustainable food system innovation. Beyond their role as sources of essential nutrients, such foods deliver biologically active constituents capable of modulating molecular signaling pathways, shaping gut microbial ecosystems, and influencing metabolic and inflammatory processes central to long-term health. In parallel, global food systems face intensifying pressures to deliver health-promoting diets while reducing environmental impact and improving resource efficiency. This Research Topic was therefore conceived to address a central and timely question: how can functional foods rich in bioactive compounds be scientifically validated, mechanistically understood, and sustainably integrated into future food systems to deliver co-benefits for human health?

This Research Topic brings together 12 peer-reviewed contributions, encompassing original research articles, narrative reviews, and a GRADE-assessed systematic review and meta-analysis. Taken together, these works signal a field in active transition—from largely descriptive cataloging of bioactive compounds toward *hypothesis-driven, mechanism-anchored, systems-level*, and *translationally oriented* research paradigms. Notably, sustainability emerges not as an auxiliary consideration but as an explicit design imperative. Across the contributions, health functionality and ecological resilience are increasingly conceptualized as interdependent and co-optimized outcomes of intentional, evidence-based food system and product design.

## From bioactive discovery to functional relevance

Several contributions address the foundational task of identifying and characterizing bioactive compounds across diverse food matrices, spanning plant-based foods, alternative biological resources, and food processing by-products. For instance, Alshehri et al. employ GC-MS and computational analysis to unveil and predict the anticancer potential of compounds in kola nut seeds. Similarly, advanced analytical approaches are central to the work of Jin et al., who purify and identify a multifunctional octapeptide from *Semen Armeniacae* glutelin-2 hydrolysates, using *in silico* screening to elucidate its restraining mechanisms against Keap1 and ACE. A consistent message emerging from these studies is that bioactivity cannot be disentangled from *food matrix, processing history*, and *physicochemical stability*, all of which shape bioaccessibility and physiological impact. This is exemplified by Huang, Zhou et al.,who study the extraction, antioxidant, and prebiotic activity of polysaccharides from *Phyllanthus emblica* L. fruits, and by Wang et al., who investigate the regulatory mechanism of fermented *Rosa roxburghii* Tratt. fruit vinegar on non-alcoholic fatty liver disease.

Complementary review articles on crops such as taro and mustard provide integrative syntheses of phytochemical composition, processing effects, and functional attributes. Tan et al. detail emerging trends in taro research, covering composition, functionality, and health benefits, while Hu and Yan offer a comprehensive review of the phytochemical components and bioactive functionality of mustard (*Brassica juncea*). These reviews highlight the dual significance of such foods: as culturally and regionally important staples, and as underexploited reservoirs of bioactive compounds with relevance to metabolic and inflammatory health. Together, these contributions reinforce the principle that effective functional foods arise from the convergence of food chemistry, processing science, and biological context, rather than from isolated compounds in abstraction.

## Mechanistic pathways and gut–host integration

A defining strength of this Research Topic is its emphasis on *mechanistic insight*. Multiple original research articles interrogate how bioactive compounds influence oxidative stress regulation (e.g., Nrf2 pathway), inflammatory signaling (e.g., NF-κb), lipid metabolism, and intestinal barrier integrity. Rahman et al. demonstrate how peptide hydrolysates from *Vespa orientalis* pupae modulate NF-κB signaling in a model of LTA-induced pneumonia, linking bioactive intake to immune regulation. The gut microbiota emerges as a critical mediator, as shown in the work of Pu et al., who explore the association of a gut microbiota dietary index with metabolic dysfunction-associated steatotic liver disease, mediated by inflammation and BMI. This theme is further supported by Yang et al. and Huang, Yang et al., who use microbiome and metabolomic insights to reveal how taurine alleviates hyperuricemia-induced nephropathy in rats. Across these studies, the gut microbiota emerges not simply as a passive target, but as an active mediator linking dietary bioactives to systemic effects across organs, including the liver, kidney, lung, and skeletal muscle.

The application of multi-omics strategies, encompassing microbiome profiling, metabolomics, and lipidomics, represents a methodological maturation of the field. Pu et al. employ hepatic lipidomics analysis to reveal the anti-obesity effects of insoluble dietary fiber combined with intermittent fasting. These tools enable a more holistic, integrated mapping of diet–microbe–host interactions and strengthen causal inference beyond single-endpoint analyses. This approach, however, also highlights persistent challenges in data integration, analytical standardization, and the biological interpretation of high-dimensional datasets, highlighting the need for continued methodological rigor.

## Functional foods in metabolic health and disease contexts

Many contributions in this Research Topic focus on metabolic health, including obesity, fatty liver disease, renal dysfunction, and exercise-associated physiological stress. The study by Zhao et al. on okara fiber and intermittent fasting is a prime example, demonstrating clear anti-obesity effects. At the level of human evidence, the inclusion of a GRADE-assessed systematic review and meta-analysis by Bideshki et al. on β-hydroxy-β-methylbutyrate (HMB) supplementation represents a critical contribution. It exemplifies best practice in evidence synthesis and reinforces the necessity of methodological discipline for advancing credible health claims. Preclinical models provide converging evidence that specific bioactive components, such as structured dietary fibers, peptides, taurine, and fermented food extracts, can beneficially modulate lipid handling, inflammatory tone, and metabolic signaling pathways. These phenotypic effects are strengthened by mechanistic data linking functional outcomes to defined molecular and microbial changes, enhancing their translational plausibility.

At the level of human evidence, the inclusion of a GRADE-assessed systematic review and meta-analysis on β-hydroxy-β-methylbutyrate supplementation represents a particularly important contribution. Beyond its specific conclusions, this work exemplifies best practice in evidence synthesis, transparency, and the critical assessment of certainty. It reinforces that the path to credible health claims and regulatory acceptance is paved with methodological discipline and rigorous evidence grading.

## Sustainability, valorisation, and food system relevance

A standout feature of this topic is how sustainability considerations are woven into the scientific narrative. Several studies demonstrate the valorization of food system side-streams, such as okara, transforming waste into sources of functional, structured fiber, and aligning health benefits with circular bioeconomy principles. Reviews on underutilized crops further argue that diversification of plant sources can enhance agrobiodiversity, regional food sovereignty, and climate resilience, while simultaneously expanding the repertoire of functional ingredients. The functional use of okara, a major soy processing by-product, as a structured dietary fiber by Zhao et al. illustrates how metabolic health benefits can be aligned with waste reduction and circular bioeconomy principles. Similarly, the crop-focused reviews by Tan et al. and Hu and Yan underscore how diversification into underutilized plant sources can support agrobiodiversity and climate resilience while expanding the repertoire of functional ingredients.

Collectively, these perspectives reinforce a critical shift in thinking: sustainability should not be treated as an external constraint on functional food development, but as a *design parameter* that shapes ingredient selection, processing strategies, and product innovation from the outset.

## Challenges and future directions

Despite substantial progress, the contributions also illuminate persistent challenges. Bioavailability, interindividual variability in response, and the translation of preclinical findings to diverse human populations remain key limitations. Furthermore, the use of complex natural extracts and fermented matrices raise additional issues related to standardization, reproducibility, and quality control. Observational associations between diet, gut microbiota, and disease risk, while informative, require confirmation through well-designed intervention studies. Future research priorities should therefore include: (i) mechanism-anchored human trials employing validated biomarkers; (ii) microbiome-informed stratification in clinical studies to decipher and predict responder heterogeneity; (iii) harmonized quality systems for complex bioactive ingredients; and (iv) life-cycle-aware product development that quantifies both health and environmental co-benefits.

Expanding the routine use of rigorous evidence-grading frameworks will be essential to guide policymakers, clinicians, industry stakeholders, and consumers alike.

## Concluding remarks

The 12 contributions assembled in this Research Topic collectively demonstrate how functional foods containing bioactive compounds can be advanced from promising concepts to scientifically grounded solutions. By integrating food chemistry, biological mechanisms, systems-level analytics, and sustainability-oriented innovation, this body of work offers a coherent and forward-looking perspective on the role of functional foods in addressing contemporary health and food system challenges.

As the field continues to mature, progress will depend not only on discovering new bioactive compounds but on designing foods that deliver them effectively, equitably, and sustainably. This Research Topic captures the momentum of this essential endeavor, powerfully underscoring the role of functional foods in building a resilient future for both human and planetary health.

